# An Act to Prevent the Sale of Drugs or Medicines Designed to Procure Criminal Abortion

**Published:** 1872-06

**Authors:** 


*An Act to Prevent the Sale of Drugs or Medicines Designed to Procure Criminal Abortion.*

SECTION I. *Be it enacted by the People of the State of Illinois, represented in the General Assembly,* That no druggist, dealer in medicines, or any other person in this State, shall sell to any person or persons any drug or medicine known or presumed to be ecbolic or abortifacient, except upon the written prescription of some well known and respectable practicing physician. And any druggist or dealer in medicines filling such prescription shall, in a book especially provided for that purpose, correctly register the name of the physician prescribing, the person to whom sold, the name of the medicine, or medicines, and the amount, together with the date of the sale.

SEC. 2. No druggist, dealer in medicine, or any other person, shall keep on hand or in any manner advertise or expose for sale, or sell, any pills, powders, drugs, or combination of drugs designed expressly for the use of females, in any other manner or form than that hereinafter described, to wit: The proprietor of any such pill, powder, drug or combination of drugs, shall submit, under oath, a true statement of the formula by which the same is compounded, to five well known and respectable practicing physicians in the county where the same is proposed to be sold; if the said physicians unanimously agree that the proposed formula is not of an abortifacient character, they shall issue their certificate, under oath, to that effect, a copy of which shall be kept, with the formula attached, for the inspection of any person desiring to see the same, by the druggist or dealer in medicine proposing or desiring to keep for sale such medicine—and this shall be his full warrant for such sale: *Provided*, this section shall not be taken or construed to apply to such compounds as are known as "Officinal."

SEC. 3. Any person or persons violating any of the provisions of this act, shall, upon conviction thereof, be punished by a fine of not less than fifty nor more than five hundred dollars, or by imprisonment in the county jail not less than thirty days or more than six months, for each and every offense, or by both.

Introduced March 9, 1871. Passed House, March 7, 1872. Passed Senate, March 22, 1872. Signed by Governor, March 27, 1872. Takes effect, July 1, 1872.

